# Role of DNA Methylation and Adenosine in Ketogenic Diet for Pharmacoresistant Epilepsy: Focus on Epileptogenesis and Associated Comorbidities

**DOI:** 10.3389/fneur.2019.00119

**Published:** 2019-02-26

**Authors:** Fan Chen, Xinghui He, Guoming Luan, Tianfu Li

**Affiliations:** ^1^Beijing Key Laboratory of Epilepsy Research, Department of Neurology, Beijing Institute for Brain Disorders, Sanbo Brain Hospital, Capital Medical University, Beijing, China; ^2^Beijing Key Laboratory of Epilepsy Research, Department of Neurosurgery, Beijing Institute for Brain Disorders, Sanbo Brain Hospital, Capital Medical University, Beijing, China

**Keywords:** ketogenic diet, epilepsy, epileptogenesis, comorbidities, adenosine

## Abstract

Epilepsy is a neurological disorder characterized by a long term propensity to produce unprovoked seizures and by the associated comorbidities including neurological, cognitive, psychiatric, and impairment the quality of life. Despite the clinic availability of several novel antiepileptic drugs (AEDs) with different mechanisms of action, more than one-third of patients with epilepsy suffer with pharmacoresistant epilepsy. Until now, no AEDs have been proven to confer the efficacy in alteration of disease progression or inhibition of the development of epilepsy. The ketogenic diet, the high-fat, low-carbohydrate composition is an alternative metabolic therapy for epilepsy, especially for children with drug-resistant epilepsy. Recently clinical and experimental results demonstrate its efficacy in ameliorating both seizures and comorbidities associated with epilepsy, such as cognitive/psychiatric concerns for the patients with refractory epilepsy. Of importance, ketogenic diet demonstrates to be a promising disease-modifying or partial antiepileptogenesis therapy for epilepsy. The mechanisms of action of ketogenic diet in epilepsy have been revealed recently, such as epigenetic mechanism for increase the adenosine level in the brain and inhibition of DNA methylation. In the present review, we will focus on the mechanisms of ketogenic diet therapies underlying adenosine system in the prevention of epileptogenesis and disease modification. In addition, we will review the role of ketogenic diet therapy in comorbidities associated epilepsy and the underlying mechanisms of adenosine.

## Introduction

Possible implications of the ketogenic diet (KD), a high-fat, low-carbohydrate diet, have been demonstrated in neurological fields, for instance: cognitive decline and dementia (1, 2), Parkinson disease (3), multiple sclerosis and its cognitive complications (4, 5), migraine and cluster headache (6–8). Epilepsy is a chronic neurological disorder characterized by a long term propensity to produce unprovoked seizures and by the associated comorbidities including neurological, cognitive, psychiatric, and impairment the quality of life. (9). Despite several novel antiepileptic drugs (AEDs) move into clinic in recent years, pharmacotherapy is not effective in 30% of all cases, and up to 30 percent of patients with epilepsy remains refractory or drug resistant (10, 11), most of them are not suitable for resective operation and have to continue to suffer from uncontrolled recurrent seizures and the lower quality of life involved an extensive range of cognitive and psychiatric symptoms. However, current AEDs have been developed for antiictogenesis (inhibition of seizures) and not for antiepileptogenesis (prevention of epilepsy or disease-modification) (12). In addition, epilepsy has been regarded as prototype neuropsychiatric illness with interface of neurology and psychiatry, and treatment of comorbidity may autonomously ameliorate the efficacy for seizures inhibition and enhance the quality of life for patients with epilepsy (13, 14). KD was developed as a non-pharmacological treatment for epilepsy, and was regarded as a last resort of therapy for children with pharmacoresistant epilepsy. The efficacy of KD in the treatment of pharmacoresistant epilepsy suggests that the mechanisms of action in controlling seizures conferred by KD are different with that of conventional AEDs (15). Clinical and experimental results indicated that KD therapy is a promising disease-modifying or partial antiepileptogenesis treatment for pharmacoresistant epilepsy (16, 17). In addition, KD therapy provides effectiveness in ameliorating both seizures and comorbidities associated with epilepsy, such as cognitive/psychiatric concerns for the patients with pharmacoresistant epilepsy (18–21), and improving the quality of life (22, 23). The satisfactory efficacy in the treatment of patients with pharmacoresistant may offer the impetus to uncover novel mechanisms underlying the development of epilepsy and associated comorbidities. Therefore, in order to develop novel therapies aim to modify the development of epilepsy (disease modifcation) and associated comorbidities, there is a critical need to strengthen the extensive research for KD from bench to bedside and bedside to bench. The present review is indicted not to offer a comprehensive overview of all potential mechanisms, but to focus on the role of KD therapy in epileptogenensis, comorbidities associated with epilepsy, as well as the possible mechanisms underlying adenosine dysfunction.

## Prevention or Modification of Epileptogenesis of the KD Therapy

The term epileptogenesis refers to a complex processes that happens prior to the initial epileptic seizure appears to translate the epileptic brain with higher propensity of recurrent seizures and processes that aggravate seizures to drug resistant (12), which involves alterations in expression and functions of receptors and ion channels, epigenetic alterations, inflammatory mechanisms, glial activation, and reorganization of neuronal circuitry (16, 24). The true antiepileptogenic efficacy means prophylactic drug treatment in prevention of spontaneous recurrent seizures after a brain insult. The term disease modification refers to the therapy may modulate the intrinsic process of the disease even though it may not hamper the occurrence of a disease (12). The halt of development of epilepsy after initial diagnosis is defined as a therapy of disease modification (12). Until now, conventional AEDs offered efficacy only for inhibition of epileptic seizures and not for prophylaxis therapeutic intervention of epilepsy or modulation of the epilepsy development. Therefore, novel avenues for ideal therapies to hamper disease development of epilepsy are imperative (16).

The high-fat, low-carbohydrate KD has been regarded as a palliative therapy for pharmacoresistant epilepsy in children and adults. The 30% of children with pharmacoresistant epilepsy on the diet had more than 90% seizure reduction, and 52% of the children had more than 50% seizure reduction at 3 months (25). Even in adult patients with drug resistant epilepsy, 32% of those using KD attained the efficacy of seizure reduction more than 50% (26). Previous data on KD therapy in adolescents and adults indicted that up to 13% of patients with pharmacoresistant epilepsy were seizure-free and approximately two thirds of patients reduced seizure more than 50% (27). Currently, most of the KD therapy studies in epilepsy mainly focused on KD efficacy in seizure control, and no valid and well-designed clinical research to evaluate the efficacy of KD therapy on antiepileptogenesis or disease modification has been implemented (16). The emerging clinical findings indicated that KD can also provide antiepileptogenic and disease-modifying therapies in pharmacoresistant epilepsy (17, 28). Some patients with KD therapy become long term seizure-free even after termination of KD therapy (17). The long term clinical efficacy in KD therapy for epilepsy over time might suggest the mechanism underlying disease-modification (29).

In addition, experimental research also indicated that KD therapy prevented disease progression in two different typical animal models of epilepsy induced by electrical kindling and chemicoconvulsants (16). In the pentylenetetrazole kindling mice model with consecutive injection of subconvulsant doses of pentylenetetrazole, KD but not a typical AEDs affords long-lasting protective efficacy (increased seizures threshold) against epileptic seizures caused by pentylenetetrazole even after termination of KD. This kindling model is an ideal animal model of epileptogenesis and has been extensively evaluated to study the pathomechanisms underlying the epileptogenic process (16). Therefore, the lasting outcomes in this kindling paradigms extensively used to evaluate the efficacy of the inherent capacity of antiepileptogenic therapy are likely to consistent with an antiepileptogenic or disease modification effect (16, 29, 30). In rats post-status epilepticus model of temporal lobe epilepsy evoked by pilocarpine, animals on the control diet displayed seizures progressed in severity and frequency, while animals on the KD diet displayed the epileptic seizures with severity and frequency significantly decreased (16). Of importance, reduction of seizures in the model maintained even after the diet reversal. Post-status epilepticus model of temporal lobe epilepsy have been extensively implanted to explore the novel AEDs with potential disease modifications, which is characterized by an first brain lesion, a dormant period, reactive astrogliosis in hippocampus and alteration of brain networks resulting in recurrent spontaneous seizures (12, 31). The results in the study highly indicated the KD offered a role in disease modification or partial antiepileptogenesis in a typical model of temporal lobe epilepsy (16).

Recent evidence demonstrated that KD treatment also confers antiepileptogenesis efficacy in genetic models of epilepsy with kcna1-null mutant mice (32, 33). Kcna1-null mutant mice model occupied with several characteristics, such as early onset epilepsy with a severe seizure phenotype with myoclonic and generalized tonic–clonic seizures, resistant to traditional AEDs, cognitively impaired, cardiac arrhythmias and sudden death (33). It is an ideal model to study the epileptogenesis or disease modification, because the mice in the model experienced several kinds of temporal lobe epilepsy syndromes, and gradually progressed into terminal events associated with human sudden unexpected death in epilepsy (33–38). KD therapy demonstrated to retard the disease development, postpone the advent of catastrophic seizures, and to increase the life span by 47% in this model of progressive epilepsy (33).

However, there exist controversies on whether KD therapy has the role of antiepileptogenesis or disease modification. In the post-traumatic epilepsy model, a good model to test antiepileptogenic therapies (39), no evidence for KD-induced antiepileptogenesis was demonstrated (40). In rats post-status epilepticus model of temporal lobe epilepsy induced by pilocarpine, another commonly used model to verify disease modification or epileptogenesis (12), KD therapy did not demonstrated to halt the clinical course of epilepsy development after status epilepticus induced by an initial lithium-pilocarpine administration (41).

In addition, the adverse effects of the KD had been reported in extensive studies (42). The chief adverse effects were gastrointestinal symptoms, such as diarrhea, obstipation, vomiting (43–46) and weight down (43, 46). Other adverse effects were also addressed, such as abdominal pain, renal stones, gallstones, infectious disease (pneumonia and sepsis), acute pancreatitis, hypercholesterolaemia, dropped bone matrix density, fatty liver, tachycardia, nephrocalcinosis, status epilepticus, acidosis, dehydration, prolonged of hospitalization, hunger and any infection of the respiratory tract (42).

## Adenosine-Dependent Epigenetic Mechanism Involving the Disease Modification Therapy of KD

Extensive experimental and clinical evidence demonstrated that disruption of glia-derived adenosine system as one of the important mechanism subserved the development of epilepsy (47), and therapeutic adenosine augmentation exerts anticonvulsant and seizure terminating efficacy (15, 47–53), mediated by both receptor-dependent and receptor–independent pathways (54). Antiictogenic effects (anticonvulsant effects) of adenosine are through adenosine receptor-dependent pathway, mainly acting via adenosine A1 receptors (A1R) (15, 48, 50, 52, 55–57). Acting through presynaptic A1R, adenosine may regulate multiple neurotransmitters releasing, and the most important inhibitory actions base on the glutamatergic system in the central nervous system (15, 58). On the other hand, acting through post-synaptic A1R, adenosine has been proved to hyperpolarize the synaptic potentials in post-synaptic neurons and boost NMDA receptor inhibition via activation of K^+^ channels (59). Our previous study demonstrated that KD increased the level of adenosine in the brain and exerted anticonvulsant effects via A1R (15).

Apart from its receptor-dependent efficacy, adenosine has been indicated to play a crucial role in modulation of DNA methylation homeostasis in receptor-independent effects (51, 54, 60). Adenosine is regarded as a mandatory end product of S-adenosylmethionine dependent transmethylation reactions (60–62). Upregulated adenosine kinase expression or deficiency of adenosine drives an increase in the transmethylation pathway leading to hypermethylated DNA, which is potentially implicated in epileptogenesis. Deficiency of adenosine and DNA hypermethylation develop into a vicious circle associated with in the onset of epileptogenesis, spontaneous seizures, progression of epilepsy and chronic pharmacoresistant epielpsy (60). Therefore, to restore the adenosine level or DNA methylation in epilepsy might be the novel and promising therapeutic target (62). Studies have demonstrated that focal augmentation of adenosine remarkably down-regulate DNA methylation in post-status epilepticus model of temporal lobe epilepsy (16). Therefore, adenosine and DNA methylation might be highlighted as emerging antiepileptogenic or disease-modification agents for epilepsy therapy (32, 62, 63).

DNA methylation has been proved to exert high fidelity modulation of gene expression in brain and play an important role in the pathogenic mechanisms of onset of epileptogenesis and development of epilepsy. Therefore, intervention of DNA methylation is regarded as a reasonable prophylaxis therapy for epilepsy in view of the fact that it acts on directly the predominant pathway that initiates the mutiple downstream cellular and molecular events mediating epileptogenesis (62). Global DNA hypermethylation has been demonstrated in patients with temporal lobe epilepsy and rats post-status epilepticus model of temporal lobe epilepsy (16, 51, 61, 62). Adenosine exerts a crucial role as an endogenous regulator of DNA methyltransferases activity. In recent study, KD therapy has been indicted to prevent disease progression via increased adenosine and decreased DNA methylation. Of note, down-regulation of DNA methylation by KD therapy maintained after diet discontinuation (16). Based on this premise, it is likely that the KD treatment plays its antiepileptogenic efficacy via an adenosine-dependent DNA methylation modulation (32).

## Comorbidities Associated With Epilepsy

The term comorbidities have been defined as “any additional distinct clinical entity” (64, 65). Several different kinds of comorbidities, such as cognitive comorbidities, psychiatric comorbidities and neurological comorbidities, exist in epilepsy (66). Psychiatric and neurological comorbidities are relatively frequent in epilepsy (67), affecting on average, 30–50% of patients (68). Currently, the goals of therapy for patients with epilepsy are not limited to reach the aim of seizure free, but must also the improvement of comorbidities associated with epilepsy, including neurological, psychiatric and cognitive comorbidities. Cognitive comorbidities include memory, attention, executive dysfunction, etc. Learning is one cognitive issue or a consequence and learning problems that lead to an major obstacle to get educational and professional success (66); Psychiatric comorbidities refer to behavior and mood problems, such as bipolar disorder, attention deficit hyperactivity disorder (ADHD), depression, anxiety disorders and autism (66). Neurological comorbidities are migraine headache (69), sleep disorders, such as sleep apnea, insomnia, restless legs syndrome, and the parasomnias (70), pain (neuropathic pain, fibromyalgia, chronic pain) and other (asthma, diabetes, and high blood pressure) (71). The comorbidities are frequent seen in patients with epilepsy, and can deteriorate quality of life further than seizures themselves do (18). Currently, the bidirectional relation between epilepsy and associated comorbidities has been paid much more attentions (72–76). Research upon the overlap of psychiatric and neurologic symptoms from a pathophysiologic and phenomenologic perspective is becoming a hot topic in epilepsy. The comorbidities associated with epilepsy are attributable to recurrent seizures and medications. In fact, the utmost recent data demonstrate that some neurocognitive and psychological comorbidities as well as structural brain changes predate the onset of seizures, with the early cognitive compromise being further magnified by the onset of epileptogenesis, and later on, by the chronicity of seizures (77–79). Therefore, the comorbidities need to be addressed in an early stage of the illness as they have a profound worse influence on the quality of life and complicate the therapeutic management of epilepsy (66). Base on this premise, it is crucial that therapy for epilepsy should aim at both seizures and comorbidities associated with epilepsy, because improving the lives of persons with epilepsy rely more on addressing comorbidities than seizures themselves (80, 81).

Even though the KD therapy has been proved to be efficacy in inhibition of seizures in patients with pharmacoresistant epilepsy, much more attention is needed to comorbidities and clinical advantages of KD. Recent study demonstrated that KD therapy afford a beneficial contribution on behavioral and cognitive function in children and adolescents with pharmacoreisistant epilepsy (18, 82, 83). On the other hand, studies with objective neuropsychiatric tests demonstrated that KD therapy afford benefits on alertness without amelioration in global cognition (20). KD therapy provided cognitive improvements in patients with pharmacoreisistant epilepsy, although it is unclear if this is an independent efficacy of the diet (84). More specifically, an improvement is observed in mood, sustained attention, and social interaction. This activation of mood and cognition was not asssociated with the decrease of seizure frequency and correlates with the prominent efficacy of the KD (19, 19, 20, 84), and appeared no relation to AEDs diminution, age when KD therapy is initiated, type of KD, and sleep amelioration (19, 20). In consistent with the effects of KD to control seizure and improve cognition in patients with pharmacoreisistant epilepsy, several lines of experimental research also demonstrated the neuroprotective efficacy on cognition (21, 85, 86). For the status of KD therapy in psychiatric comorbidities associated epilepsy, such as depression or bipolar disorder, although currently there is no long-term, prospective, randomized, placebo-controlled crossover dietary clinical trial (87), KD has been regarded as a novel frontier therapy for mood disorder, particularly in therapy with drug resistant mood disorder (88).

KD therapy has a long been used in children with pharmacoresistant epilepsy. However, overall distinct role of KD in comorbididties associated with epilepsy, especially psychiatric comorbididties is unclear. The impact of KD in psychiatric disorders is speculative. There is no solid evidence to corroborate this statement. Currently, there is inadequate evidence for the administration of KD in psychiatric comorbidities associated with epilepsy including behavior and mood problems, such as attention deficit disorder, bipolar disorder, depression, anxiety disorders, schizophrenia, autism spectrum disorder, and combinations of these conditions (87). So far, KD therapy is still not a recommended treatment option for the psychiatric disorders.

## Adenosine Dysfunction in Comorbidities Associated With Epilepsy

For the cognitive comorbidities, extensive clinical and experimental study has been proved that adenosine plays an important role in controlling inflammation inhibiting seizures (15, 47–50, 52, 53, 55, 56, 89), and regaining cognitive function when cognition is afflicted secondary to epilepsy (47, 52, 53, 60). Adenosine impacts cognition processes via action on adenosine receptor A2A (A2AR) signaling pathway and regulation of neurotransmitters including glutamatergic, dopaminergic, GABAergic, and BDNF (90). The astrocytic A2AR might play a prominent role in interacts with glutamate transporter-1 and thereby regulates astroglial glutamate uptake. Therefore, malfunction of A2AR in astrocytes, by moduating glutamate transporter-1activity, initiates an astrocyte-to-neuron wave of communication causing disruption of glutamate system and cognitive impairment (91). Increasing the adenosine level in the brain via pharmacologic inhibition of the key enzyme of adenosine clearance, or intrastriatal implants of engineered adenosine-releasing cells can improve cognitive function (92). KD therapy has been proved to increase the adenosine level in the brain (15, 16), and KD therapy has been demonstrated to afford an improvement of cognitive activation in the patients with epilepsy (19). Based on the evidence above, it is strongly suggested that KD therapy ameliorated the cognition deficit though augmentation of the adenosine in the brain, which might be highly regarded as an ideal method for the therapy of cognitive comorbidities associated with epilepsy.

Depression is the common psychiatric comorbidities in patients with epilepsy (93). A1R signaling pathway in astrocytes has been demonstrated to be necessary in decreasing depressive-like behaviors secondary to sleep deprivation in mice (90), which indicated the activation of adenosine signaling triggered by sleep deprivation contributes to inhibition of depression (94). S-adenosylhomocysteine, a precursor of adenosine, has been found to be efficacy for the patients with treatment-resistant depressive disorder (95). The common clinical antidepressive treatment, such as acupuncture (96, 97) and deep brain stimulation (98) has been indicated via increasing of adenosine and activation of A1R in the brain (99–101). KD therapy, the adenosine augmentation approach (15, 16), has been demonstrated to be effective in inhibition of depression (87). Therefore, it is suggested that one of the mechanisms underlying the KD therapy for depression via A1R. KD therapy might represent a novel strategy for the therapy of psychiatric comorbidities associated epilepsy.

Sleep disorders is the common neurologic comorbidities associated with epilepsy (70). Extensive studies have demonstrated that adenosine plays a crucial role in modulation of sleep homeostasis (90, 102, 103). Adenosine receptors A1R and A2AR have been demonstrated to play an important role in sleep modulation (90). A1R agonists promote sleep (104), while A1R antagonists inhibit sleep (105), via basal forebrain mediated mechanisms, respectively. A2AR agonist also has been demonstrated to promote sleep (106), via activation of cells of the leptomeninges or nucleus accumbens to reinforce the neuronal activity in ventrolateral preoptic region (107). This effect of sleep regulation was not found in A2AR-deficient mice (108). Inhibition of the A2AR in the shell of the nucleus accumbens (90, 109) has been reputed the potential mechanism of the arousal effects of caffeine (non-selective antagonist of A1R and A2AR).

Ii is well-accepted that of upregulation of adenosine (increasing adenosine level or activation of adenosine receptors) promote sleep and downregulation of adenosine (decrease adenosine level or inactivation of adenosine receptors) induce wakefulness (90). It is hypothesized that a decreased adenosine tone uniformly forms the base for both epilepsy and sleep disruption (90). However, recent study found a lineal association of regionally distinguishable dichotomous levels of adenosine in one model represent both epilepsy and comorbid sleep disorders (110). In the model, adenosine level was decreased in the dorsal hippocampus contributing to seizure threshold diminution, while adenosine level was increased in the lateral hypothalamus leading to chronic partial sleep deprivation. To clarify the specific brain regional alterations in adenosine tone in patients underlies both epilepsy and sleep disorder is important for the targeted therapy in the future.

## Author Contributions

The overall review design was conceived and supervised by TL. FC, XH, and GL helped in the writing different parts of the review. All authors read and approved the final manuscript.

### Conflict of Interest Statement

The authors declare that the research was conducted in the absence of any commercial or financial relationships that could be construed as a potential conflict of interest.
